# The histone demethylase *KDM3A* regulates the transcriptional program of the androgen receptor in prostate cancer cells

**DOI:** 10.18632/oncotarget.15681

**Published:** 2017-03-03

**Authors:** Stephen Wilson, Lingling Fan, Natasha Sahgal, Jianfei Qi, Fabian V. Filipp

**Affiliations:** ^1^ Systems Biology and Cancer Metabolism, Program for Quantitative Systems Biology, University of California Merced, Merced, CA, USA; ^2^ Department of Biochemistry and Molecular Biology, Marlene and Stewart Greenebaum Comprehensive Cancer Center, University of Maryland School of Medicine, Baltimore, MD, USA; ^3^ Centre for Molecular Oncology, Barts Cancer Institute, Queen Mary University of London, London, United Kingdom

**Keywords:** cancer systems biology, epigenomics, ChIP-Seq, oncogene, prostate cancer

## Abstract

The lysine demethylase 3A (*KDM3A*, JMJD1A or JHDM2A) controls transcriptional networks in a variety of biological processes such as spermatogenesis, metabolism, stem cell activity, and tumor progression. We matched transcriptomic and ChIP-Seq profiles to decipher a genome-wide regulatory network of epigenetic control by KDM3A in prostate cancer cells. ChIP-Seq experiments monitoring histone 3 lysine 9 (H3K9) methylation marks show global histone demethylation effects of KDM3A. Combined assessment of histone demethylation events and gene expression changes presented major transcriptional activation suggesting that distinct oncogenic regulators may synergize with the epigenetic patterns by KDM3A. Pathway enrichment analysis of cells with *KDM3A* knockdown prioritized androgen signaling indicating that *KDM3A* plays a key role in regulating androgen receptor activity. Matched ChIP-Seq and knockdown experiments of *KDM3A* in combination with ChIP-Seq of the androgen receptor resulted in a gain of H3K9 methylation marks around androgen receptor binding sites of selected transcriptional targets in androgen signaling including positive regulation of *KRT19*, *NKX3-1*, *KLK3*, *NDRG1*, *MAF*, *CREB3L4*, *MYC*, *INPP4B*, *PTK2B*, *MAPK1*, *MAP2K1*, *IGF1*, *E2F1*, *HSP90AA1*, *HIF1A*, and *ACSL3*. The cancer systems biology analysis of KDM3A-dependent genes identifies an epigenetic and transcriptional network in androgen response, hypoxia, glycolysis, and lipid metabolism. Genome-wide ChIP-Seq data highlights specific gene targets and the ability of epigenetic master regulators to control oncogenic pathways and cancer progression.

## INTRODUCTION

Methylation of histone lysine residues is a significant component of epigenetics and is associated with control of gene expression [1]. Specifically, methylation of lysine 9 of histone H3 (H3K9) has been recognized as hallmark of transcriptionally suppressed genes [2]. *KDM3A* (lysine demethylase 3A; Gene ID: 55818; also referred to as JMJD1A or JHDM2A) is crucial for gene regulation in a variety of biological activities such as spermatogenesis, metabolism, stem cell activity and tumor progression by demethylating mono- or di-methylated H3K9 [3–5]. Although the KDM3A protein regulates a wide array of target genes in tissue- and development-specific settings, chromatin modifiers often lack intrinsic DNA sequence specificity. Therefore, how KDM3A is targeted to specific genes is an area of current research interest and important for understanding epigenetic dysregulation in human disease.

KDM3A activity is deregulated in several cancers [3, 6–8]. In prostate adenocarcinoma (PRAD), KDM3A functions as a transcriptional coactivator for the androgen receptor (*AR*; Gene ID: 367) [3, 9]. The ability to cooperate with the AR highlights a potential role of KDM3A as coactivator and driving force for sex-specific tissue development as well as for prostate cancer initiation and progression. In PRAD, androgen-dependent signaling plays a key role in the oncogenesis of prostate epithelial cells and the aggressiveness of the malignancy [10, 11]. The AR transcription factor belongs to the nuclear receptor superfamily and contains a C-terminal ligand-binding domain. Upon ligand binding, the AR undergoes a conformational change and dissociates from a cytosolic chaperone protein complex. Its ligand-bound conformation allows the AR to dimerize and to translocate into the nucleus [12]. Once in the nucleus, the activated AR dimer binds to androgen response elements (AREs) present in the promoter or enhancer of AR-regulated target genes and recruits co-activators or co-repressors to regulate gene expression [13]. In addition to the AR, KDM3A has been found to regulate expression and/or activity of several transcription factors such as *PPARG*, *KLF2*, *ESR1*, and *HOXA1* [14–17].

In order to further elucidate the impact of *KDM3A* on the epigenome, we performed chromatin immunoprecipitation in combination with next generation sequencing (ChIP-Seq) of its binding and demethylation activity. We quantified changes of H3K9me1 or H3K9me2 marks, the two substrates of KDM3A, and mapped AR-binding in the CWR22Rv1 prostate cancer cell line in combination with knockdown of *KDM3A*. Alteration of H3K9 methylation marks mapped to genomic locations coinciding with AR binding pinpoints target genes and oncogenic pathways cooperatively regulated by KDM3A and AR.

## RESULTS

### Genomic annotation and transcriptional regulation of KDM3A specific demethylase activity

**Figure 1:**
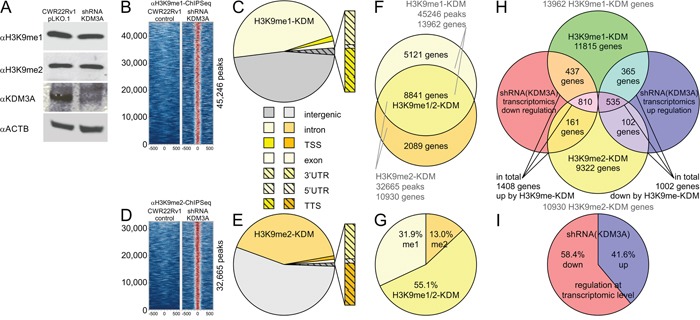
ChIP-Seq experiments with matched knockdown of *KDM3A* show gain of histone 3 lysine 9 methylation and transcriptional deactivation **A**. Western blot of prostate cancer line CWR22Rv1 with antibodies against H3K9me1, H3K9me2, KDM3A (lysine demethylase 3A; Gene ID: 55818; also referred to as JMJD1A or JHDM2A), and beta-actin. ChIP-Seq experiments using **B-C**. H3K9me1-antibody and **D-E**. H3K9me2-antibody show specific gain of signal following small hairpin RNA (shRNA) knockdown of the histone methyltransferase *KDM3A* in the prostate cancer line CWR22Rv1. Genomic location of C) H3K9me1 and E) H3K9me2 sites identified by ChIP-Seq. **F**. H3K9me1 and H3K9me2 ChIP-Seq signals are overlapped and annotated. **G**. The majority of histone demethylase events due to KDM3A activity is detected by both, H3K9me1 and H3K9me2, ChIP-Seq antibodies. **H**. Overlay of gene mapping of histone methylation events identified by ChIP-Seq and transcriptomics experiments. Using this data we defined the group of 1408 genes as positively regulated by KDM3A activity (down in the prostate cancer line CWR22Rv1 with shRNA knockdown of *KDM3A*), and 1002 genes as negatively regulated by KDM3A activity. **I**. Transcriptomic impact of *KDM3A* knockdown shows 58.4% of gene activation (down in the prostate cancer line CWR22Rv1 with shRNA knockdown of *KDM3A*), and 41.6% of gene silencing.

Knockdown of *KDM3A* in CWR22Rv1 cells showed minor effects on global levels of H3K9me1 or H3K9me2 by Western blot analysis (Figure 1A), suggesting that KDM3A demethylates a small pool of methylated histone marks and regulates a specific set of gene targets. In order to establish the genome-wide impact of the epigenetic regulator KDM3A, we conducted a matched ChIP-Seq experiment using antibodies specific for histone marks H3K9me1 and H3K9me2 in combination with small hairpin RNA (shRNA) knockdown of *KDM3A* in the CWR22Rv1 cell line (Supplementary Table 1). Histone lysine demethylation (KDM) events mediated by KDM3A were defined by gain of methylation ChIP-Seq signals following knockdown of *KDM3A* (Figure 1B–1E). Alterations of H3K9me1 and H3K9me2 histone marks upon *KDM3A* knockdown were evaluated in comparison to reference genomic DNA input or control non-coding shRNA samples. Overall, the peak counts of both H3K9me1 and H3K9me2 ChIP-Seq experiments showed a gain of signal (32244 to 34162 and 23353 to 46599, respectively). Since H3K9 methylation is a mark associated with the highly condensed heterochromatin state, we characterized the specific genomic regions associated with both H3K9 histone methylation marks. KDM events were functionally annotated by mapping bound regions to the human genome and by classifying them according to the nearest gene locus and relative position within coding regions. Promoter or transcription start sites (TSS) and transcription termination sites (TTS) genomic annotations are defined as being within ± 1000 bp of the gene-coding body. Intergenic regions were defined as the remaining regions outside the gene body. In the H3K9me1 ChIP-Seq experiment the intergenic regions were the most frequently found region with 21112 peaks (46.5%) followed by 21495 (47.7%) as intronic regions, 822 (1.8%) as exonic regions, 606 (1.3%) as promoter-TSS regions, 549 (1.2%) as TTS regions, 424 (0.9%) as 3′UTR regions, and 46 (0.1%) as 5′ untranscribed (UTR) regions (Figure 1C). Similarly, the H3K9me2 ChIP-Seq had the intergenic region as its most frequent region with 18195 (55.9%) followed by 13167 (55.9%) as intronic regions, 373 (1.1%) as exonic regions, 355 (1.1%) as promoter-TSS regions, 246 (0.8%) as TTS regions, 204 (0.6%) as 3′UTR regions, and 25 (0.1%) as 5′ UTR regions (Figure 1E). Taken together, ChIP-Seq profiles monitoring histone 3 lysine 9 methylation marks following *KDM3A* knockdown revealed selective histone demethylation effects of this epigenetic modifier.

Following genomic annotation, we were curious if there was a specific gene expression program underlying demethylation of H3K9me1 and H3K9me2. Half of the detected genes, 8841 (55.1%), contain both H3K9me1/2 marks (Figure 1F–1G). While the ChIP-Seq data shows that H3K9me1 has more annotated genes compared to H3K9me2 (5121 and 2089 respectively), both histone marks showed an equal fraction of genes being transcriptionally responsive to *KDM3A* knockdown according to the transcriptomic dataset. Overall, from the transcriptomic experiments, 1408 (58.4%; using a significance cutoff with adjusted p-values below 0.05) genes are reported as differentially down-regulated upon shRNA knockdown of *KDM3A* while 1002 genes are reported as up-regulated by KDM3A knockdown (Figure 1H–1I). The combination of ChIP-Seq histone demethylation events and transcriptomic assessment showed major transcriptional activation by KDM3A, suggesting that KDM3A may synergize with distinct transcriptional regulators for epigenetic control of gene expression.

### Identification of an epigenetic and transcriptional network in androgen receptor signaling regulated by KDM3A

**Figure 2:**
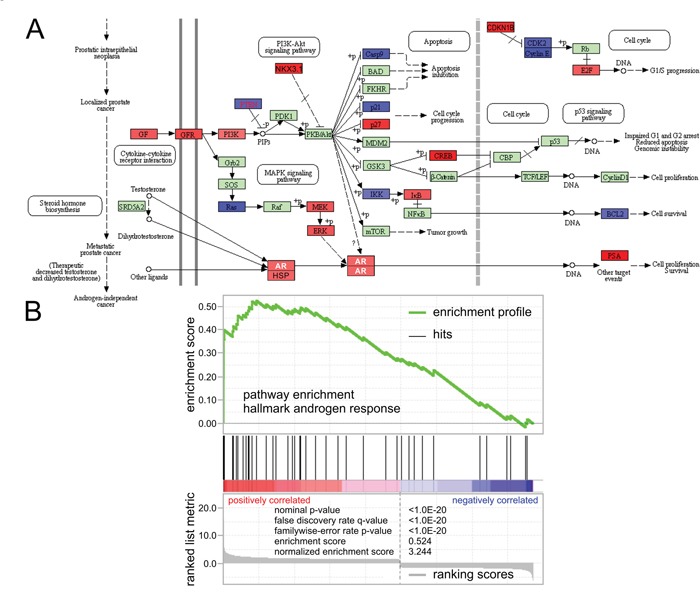
Knockdown of *KDM3A* results in epigenetic control and transcriptional activation of the androgen response **A**. Map of transcriptional regulation of KDM3A on androgen signaling. Red indicates positive response of *KDM3A* on gene (down with shRNA knockdown of *KDM3A*); blue indicates negative response of *KDM3A* on gene (up with shRNA knockdown of *KDM3A*). **B**. Gene set enrichment analysis of ranked transcriptomic data upon shRNA knockdown of *KDM3A* indicated significant enrichment of hallmark gene set of androgen response with p-value and false discovery rate q-value below 1.0E-20.

**Figure 3:**
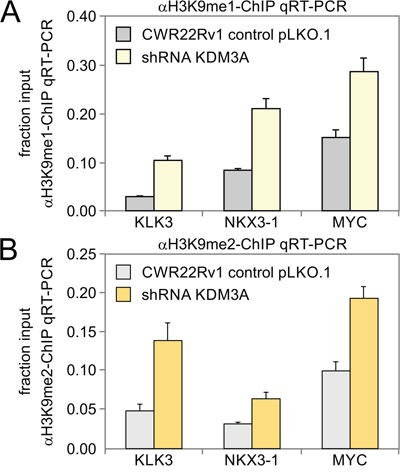
Detection of histone demethylase events by ChIP-qRT-PCR due to KDM3A activity in target genes involved in the androgen response CWR22Rv1 prostate cancer cells transfected with pLKO.1 control or *KDM3A* shRNA were subjected to the chromatin immunoprecipitation coupled with quantitative real time polymerase chain reaction (ChIP-qRT-PCR) assay using immunoprecipitation with **A**. H3K9me1-antibody and **B**. H3K9me2-antibody. The precipitated chromatin fragments were analyzed by qRT-PCR using oligonucleotides for identified androgen response element regions of *KLK3*, *NKX3-1* or *MYC*.

Following characterization of histone H3K9me1/2 marks we determined enrichment of transcriptional motifs associated with these histone marks controlled by KDM3A in prostate cancer cells. The goal of this analysis is to identify potential transcription factors that cooperate with KDM3A to regulate gene expression. Using the Jaspar motif database, we conducted an unbiased search for significant enrichment of transcription factor families (analysis of motif enrichment search with p-values below 0.05). Top hits included the androgen receptor, sterol regulatory element binding factor (SREBF), hypoxia inducible factor (HIF), activator protein 1 (AP1) complex of JUN/FOS, Krüppel-like factors (KLF), v-myc avian myelocytomatosis viral oncogene homolog (MYC), and forkhead box (FOX) families of transcription factors with significant enrichments and p-values below 1.0E-04 (Table 1). In addition, we enhanced simple ChIP-Seq-based searches with position-specific matrices to determine which transcription factor motifs were enriched compared to shuffled background sequences. The enrichment analysis showed the androgen response element (ARE) with 2915 incidences as one of the most frequent motifs detected (Table 1). Next, we analyzed significantly altered expression levels upon *KDM3A* shRNA knockdown (Figure 2A). At the transcriptional level, the androgen response gene set was the most enriched with a p-value and false discovery rate q-value each below 1.0E-20 (Figure 2B). Next, we sought inferred transcriptional regulators by comparing transcriptional targets to datasets that outline targets of transcription factors through the use of Ingenuity Pathway Analysis. The transcription factors AR, HIF, MYC, and AP1 complex were significantly enriched with p-values below 1.0E-07. Lastly, we merged ChIP-Seq profiles of H3K9Me1/2 and KDM3A transcriptional data focusing on 1408 annotated genes (overlap of H3K9ME1/2 ChIP-Seq with transcriptomic data that were down-regulated upon shRNA *KDM3A* knockdown) (Figure 1I, Supplementary Table 2). The data contained the highest enrichment ratio (26.7%) in a significantly enriched set of 27 genes in androgen signaling with p-values below 1.0E-17 and q-values below 1.0E-15 (Supplementary Table 3). In detail, putative KDM3A-regulated genes included pathways involved in androgen response, androgen receptor signaling, androgen biosynthesis, prostate cancer, pathways in cancer, cholesterol homeostasis, bile acid metabolism, aldosterone-regulated reabsorption, and progesterone regulation, hinting at the possibility of hormone nuclear steroid receptor involvement (enrichment with p-values below 2.62E-02 and q-values below 9.35E-02 correcting for multiple hypotheses testing) (Table 2). Interestingly for the concept of cooperative control, the pathways of regulation and coregulation of androgen receptor activity were also enriched with p-values and q-values below 4.04E-06 and 1.97E-04, respectively. *SLC26A2*, *FKBP5*, *KRT19*, *SORD*, *HOMER2*, *NDRG1*, *TPD52*, *INPP4B*, *PTPN21*, *ZMIZ1*, *PMEPA1*, *PPAP2A*, *TSC22D1*, *ACSL3*, *KLK3*, *NKX3-1*, *ELL2*, *MAP7*, *PTK2B*, *SMS*, *SPDEF*, *ABCC4*, *KLK2*, *MAF*, *TARP*, *AZGP1*, and *TMPRSS2* were key regulators of prostate cancer and AR signaling based on the KDM3A ChIP-Seq data (Figure 2A–2B). Transcriptional control of key players of cancer and AR signaling by KDM3A such as *KLK3*, *NKX3-1*, *MYC* were validated by chromatin immunoprecipitation coupled with quantitative real time polymerase chain reaction (ChIP-qRT-PCR) (Figure 3A–3B). Taken together, complementary analyses identified strong transcriptional networks including AR, MYC, FOX, KLF, AP1, and SREBF transcription factors that may be regulated by KDM3A. Androgen signaling was consistently identified by all of these different enrichment approaches, suggesting a key role for KDM3A in regulating AR activity.

**Table 1:** Occurrence and enrichment by Fisher's exact test reveals enrichment of transcription factor motifs in KDM3A ChIP-Seq data

transcription factor	motif occurrences	adjusted p-values of motif enrichment H3K9me1 ChIP-Seq	adjusted p-values of motif enrichment H3K9me2 ChIP-Seq
TP53	3536	4.79E-10	5.58E-14
AR	2915	7.14E-07	2.00E-05
SREBF	1969	7.98E-50	8.24E-14
HIF	1964	1.88E-179	3.06E-142
FOS	1504	2.85E-256	2.60E-123
KLF	1244	4.06E-110	5.47E-81
MYC	767	1.68E-75	4.12E-63
FOX	584	0.00E-00	4.50E-276

Adjusted p-values accounting for multiple hypotheses testing using the false discovery rate controlling procedure of Benjamini and Hochberg of transcriptional motifs below 0.05 in H3K9me1/2-KDM3A ChIP-Seq data were considered significant.

**Table 2:** Enrichment of androgen-related signaling pathways in H3K9me1/2-KDM3A ChIP-Seq data

pathway	network	K	k	k/K	p-value	q-value
androgen response	hallmarks	101	27	0.267	2.77E-18	1.63E-16
pathways in cancer	kegg	328	36	0.110	4.49E-11	8.15E-10
androgen-mediated signaling	commons	130	21	0.162	1.58E-09	1.35E-07
regulation of androgen receptor activity	commons	108	17	0.157	8.25E-08	5.28E-06
cholesterol homeostasis	hallmarks	74	12	0.162	2.36E-06	1.69E-05
bile acid metabolism	hallmarks	112	14	0.125	8.35E-06	4.87E-05
aldosterone regulated sodium reabsorption	kegg	42	8	0.191	3.43E-05	1.42E-04
bladder cancer	kegg	42	8	0.191	3.43E-05	1.42E-04
coregulation of androgen receptor activity	commons	61	11	0.180	4.04E-06	1.97E-04
prostate cancer	kegg	89	11	0.124	8.43E-05	2.93E-04
colorectal cancer	kegg	62	9	0.145	1.05E-04	3.54E-04
androgen receptor signaling pathway	wiki	91	14	0.154	1.50E-06	8.35E-04
nongenotropic androgen signaling	commons	26	5	0.192	1.40E-03	1.58E-02
validated nuclear hormone receptor network	commons	65	7	0.108	5.20E-03	3.45E-02
progesterone-mediated signaling	kegg	86	8	0.093	7.10E-03	3.79E-02
bile acid secretion	kegg	71	7	0.099	8.40E-03	3.86E-02
bile acid biosynthesis	kegg	16	3	0.188	1.41E-02	4.68E-02
bile acid metabolism	commons	27	4	0.148	1.09E-02	5.33E-02
SREBF in cholesterol and lipid homeostasis	wiki	16	3	0.188	1.41E-02	7.96E-02
androgen biosynthesis	commons	8	2	0.250	2.62E-02	8.25E-02
cholesterol biosynthesis	wiki	18	3	0.167	1.96E-02	9.35E-02

The analysis is based on a query set of 1408 genes positively regulated by KDM3A with coincidence of demethylation and transcriptomic regulation (compare Figure 1). P-values below 0.05 from the hypergeometric distribution for K, the number of elements in the pathway of interest, k, the number of elements in the intersection of the input gene set, N=45956, all known human genes, and n=1408, the number of elements in the input gene set and false discovery rate q-values below 0.10 for multiple hypotheses testing according to the controlling procedure of Benjamini and Hochberg were considered significant.

### Matched KDM and AR ChIP-Seq experiments reveal coincidence of demethylase binding, demethylation and AR binding events

**Figure 4:**
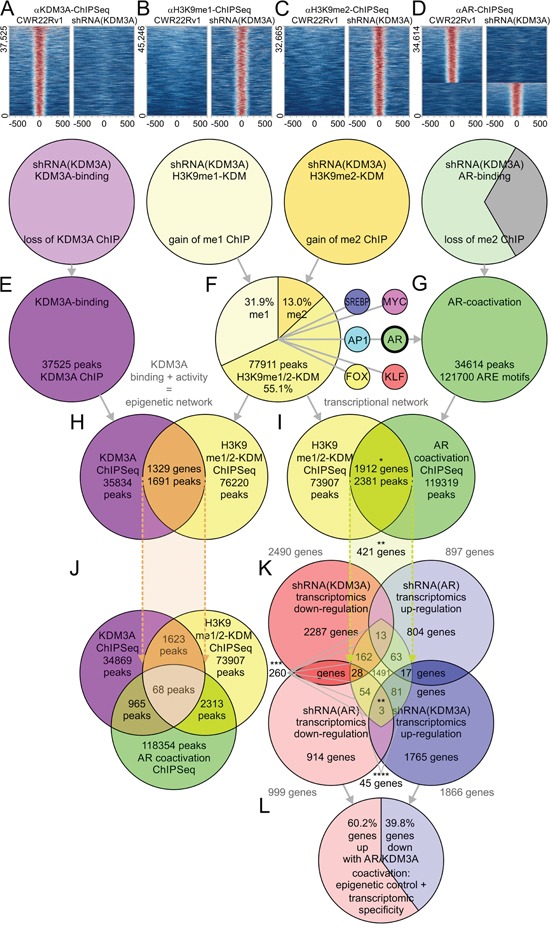
Matched ChIP-Seq and knockdown experiments of *KDM3A* in combination with ChIP of the androgen receptor show synergy of KDM3A and the androgen receptor ChIP-Seq experiments in combination with *KDM3A* knockdown results in **A**. specific loss of KDM3A ChIP-Seq signal, **B**. specific gain of H3K9me1 ChIP-Seq signal for 97.1% of the observed histone marks, **C**. specific gain of H3K9me2 ChIP-Seq signal for 95.6% of the observed histone marks, and **D**. specific loss of ChIP-Seq signal for the androgen receptor (AR) with less than 2.3% retained binding. Peak calling utilizing a model-based analysis of ChIP-Seq algorithm results in **E**. 37525 peaks for KDM3A binding, **F**. 77911 peaks for H3K9 demethylation events (H3K9me1/2-KDM), and **G**. 34614 peaks for matched AR binding including 121700 androgen response elements (AREs). Motif analysis of KDM3A ChIP-Seq signals identifies pattern of transcription factor families including the AR motivating analysis of epigenetic and transcriptional cooperation between KDM3A and AR. **H**. KDM3A and H3K9 methylation ChIP-Seq signals are overlapped and annotated. **I**. H3K9 methylation and AR ChIP-Seq signals are overlapped and annotated. 1912 genes (gene set marked with *) showed coincidence of demethylation and AR binding events. **J**. Overlap of KDM3A and H3K9 methylation ChIP shows strong epigenetic network of KDM3A binding and activity. Overlap of KDM3A, H3K9 methylation, and matched AR ChIP shows participation of KDM3A in transcriptional activation. **K**. 421 genes (gene set marked with **) showed transcriptomic change upon *KDM3A*/*AR* knockdown in addition to coactivation detected by KDM/AR ChIP-Seq. 260 genes (gene set marked with ***) were positively regulated by KDM3A or AR activity (down in the prostate cancer line CWR22Rv1 with shRNA knockdown of *KDM3A*) and identified by KDM/AR ChIP-Seq. 45 genes genes (gene set marked with ****) showed overlap of transcriptomic response upon *KDM3A* and *AR* knockdown as well as coactivation detected by KDM/AR ChIP-Seq. **L**. Transcriptomic impact of *KDM3A* knockout shows 60.2% of gene activation (down in the prostate cancer line CWR22Rv1 with shRNA knockdown of *KDM3A*), and 39.8% of gene silencing.

Knockdown of *KDM3A* in CWR22Rv1 cells resulted in loss of KDM3A ChIP-Seq binding accompanied by specific, matched gain of histone lysine 9 demethylation (Figure 4A–4C). Knockdown of *KDM3A* had little effect on the protein level of AR [3, 18]. We examined the alteration of AR binding by ChIP-Seq with an AR antibody following *KDM3A* knockdown and quantified the overlap of AR ChIP-Seq events with KDM3A binding and changes in epigenetic H3K9me1/2 marks (Figure 4A–4D). The activity-based ChIP-Seq array matched with knockdown of *KDM3A* resulted in 37525 peaks associated with KDM3A binding, 45246 and 32665 H3K9 mono- and di-demethylation (H3K9me1/2-KDM) events, respectively, and 34614 peaks for KDM3A-matched AR binding. Such an experimental design allows one to distinguish between histone demethylase binding (Figure 4E), epigenetic activity (Figure 4F), and coactivator binding events (Figure 4G). Gain of H3K9me1/2 was coupled to specific changes in AR binding in the *KDM3A* knockdown experiments (Figure 1A–1E). Overall 37.0% of the AR ChIP-Seq peaks with altered H3K9me1/2 signal were suppressed upon knockdown of *KDM3A*, while the remaining fraction was not affected. The genome-wide ChIP-Seq analysis is consistent with the biochemical data, demonstrating that KDM3A in effect recruited AR to target genes [3, 18]. KDM3A ChIP-Seq and H3K9me1/2-KDM ChIP-Seq in combination with matched knockdown of *KDM3A* produced an epigenetic network that overlaid with the AR ChIP-Seq data (Figure 4H–4J). In the case of matched and merged datasets of AR ChIP-Seq in combination with *KDM3A* knockdown, we identified in total 77911 H3K9me peaks (Figure 4F) and directly overlaid them with 34614 AR peaks containing 121700 ARE motifs (Figure 4I). Importantly, using such matched ChIP-Seq analyses, a set of 1912 genes was identified that showed an overlap of demethylation and AR binding events (KDM3A/AR ChIP, 2381 peaks, 1912 genes) (Figure 4I, Supplementary Table 1). Epigenetic events identified by ChIP-Seq were overlaid with transcriptomic data, defining a set of 421 genes that had epigenetic marks (H3K9me1/2 ChIP), AR binding (AR ChIP), and a transcriptomic effect (differential expression in either *KDM3A* or *AR* knockdown experiments) (Figure 4K, Supplementary Table 4). Similar to the initial gene set based exclusively on H3K9me1/2 marks (57.2%) (Figure 1), 60.2% of genes scored as activated upon KDM3A/AR coactivation while 39.8% were silenced. Merged ChIP-Seq data of KDM3A/AR coactivation with transcriptomic data of *KDM3A* knockdown defined a set of 260 genes (whereas activation is interpreted as down-regulation upon shRNA knockdown) (Figure 4K–4L, Supplementary Table 4).

### KDM3A and AR coactivation results in oncogenic pathway activation of AR signaling

H3K9 demethylation is known for stimulating gene expression [3, 5, 19, 20]. We used gene set enrichment analysis to identify signaling networks or functional clusters of genes controlled by both KDM3A and AR. At the gene level we studied pathway enrichment for the following three sets: A) 1912 genes defined by overlapping H3K9 demethylation/AR ChIP (Figure 4I, Supplementary Table 1-2); B) 421 genes defined by overlapping H3K9 demethylation/AR ChIP with differential expression upon *KDM3A*/*AR* knockdown (Figure 4K, Supplementary Table 2); and C) 260 genes that are down-regulated upon *KDM3A* or *AR* knockdown and having overlapping H3K9 demethylation/AR ChIP (Figure 4K, Supplementary Table 2). The gene sets were designed in a hierarchical fashion such that the parental set A of 1912 genes includes subset B of 421 genes, and B includes subset C of 260 genes (Supplementary Table 2). Such a hierarchical structure of gene sets allows one to monitor and identify requirements and conservation of a functional outcome. The gene set enrichment analysis revealed oncogenic activation of androgen signaling and metabolic pathways with p-values below 1.40E-02 and q-values below 4.44E-02 in hypoxic response, glycolysis, and lipogenic metabolism (Table 3, Supplementary Table 5). Enrichments of androgen response, metabolic pathways, hypoxia, aldosterone-regulated sodium reabsorption, glycolytic and glycerophospholipid metabolism pathways were conserved and enriched from the overlapping H3K9 demethylation/AR ChIP (1912 genes) to the genes displaying down-regulation upon *KDM3A*/*AR* knockdown and overlapping H3K9 demethylation/AR ChIP signal (260 genes). For example, androgen response and metabolic pathways showed incrementally higher enrichment with better defined input gene sets (significant enrichment of genes in androgen response with p-value of 7.51E-07 for 1912 gene set and decreased p-value of 5.23E-09 for 260 gene set). Similarly, pathways of glycolytic and glycerophospholipid metabolism showed consistently higher enrichment with the lowest p-values in the set of 260 genes, indicating that KDM3A demethylation targets these pathways and causes an up-regulation of gene expression. Of all genes detected by both H3K9 demethylation and AR ChIP-Seq experiments as well as *KDM3A* and *AR* differential expression following knockdown (core set of 45 genes; 28 genes up-regulated; 17 genes down-regulated), the AR response was the only significantly enriched pathway with a p-value of 5.29E-08. 7 genes, *NDRG1*, *PTK2B*, *ACSL3*, *KRT19*, *INPP4B*, *NKX3-1*, and *MAF*, with direct implication in AR signaling were present in all gene sets of the hierarchical enrichment analyses, thereby subject to KDM3A control by H3K9 demethylation, AR binding, and differential expression following knockdown (Supplementary Table 1-3, 5). In the androgen response pathway KDM3A and AR had strong ChIP-Seq activity (Table 4). The majority of epigenetic H3K9 demethylation events resulted in up-regulation of target gene activity and occurred at the TSSs of target genes. In addition, multiple introns, exons, and TTS of target genes were also implicated in KDM3A control. A network upstream regulator analysis implicated the AR to coordinate with epigenetic and transcriptional responses observed upon knockdown of *KDM3A* (Supplementary Table 6). At the transcriptional level, KDM3A may affect AR signaling directly by interacting with regulatory regions of *HSP90AA1* and *AR* genes. In addition, downstream effects of AR signaling were observed at *KRT19*, *NKX3-1*, *KLK3*, *TMPRSS2*, *PMEPA1*, *NDRG1*, *MAF*, *CREB3L4*, *MYC*, *INPP4B*, *PTK2B*, *MAPK1*, *MAP2K1*, *IGF1*, *E2F1*, *HIF1A*, *TARP*, *FKBP5*, *SPDEF*, *SMS*, *PPAP2A*, *SEPP1*, *UAP1*, *SORD*, *AZGP1*, *BCL2L1*, *ACSL3*, *CHUK*, and *CDKN1A* (Table 4).

**Table 3:** Pathway enrichment of transcriptional coactivation and matched ChIP-Seq experiments of H3K9me1/2 marks and the androgen receptor

pathway	network	1912 gene set (*)		421 gene set (**)		260 gene set (***)	
		p-value	q-value	p-value	q-value	p-value	q-value
androgen response	hallmarks	7.51E-07	1.27E-05	3.13E-07	2.46E-05	5.23E-09	1.23E-06
metabolic pathways	kegg	2.58E-06	1.55E-02	6.51E-09	6.00E-05	3.78E-07	1.80E-03
hypoxia	hallmarks	1.14E-11	8.99E-10	4.14E-10	9.77E-08	1.84E-06	2.17E-04
TNFα signaling via NFκB	hallmarks	1.96E-06	2.72E-05	1.34E-05	3.96E-04	1.28E-04	7.17E-03
estrogen response early	hallmarks	6.77E-09	2.66E-07	8.17E-05	1.48E-03	1.28E-04	7.17E-03
protein secretion	hallmarks	3.83E-07	8.22E-06	2.02E-07	2.38E-05	1.94E-04	7.63E-03
galactose metabolism	kegg	1.23E-03	5.79E-03	1.54E-03	1.65E-02	3.86E-04	1.30E-02
leukocyte transendothelial migration	kegg	1.04E-04	6.79E-04	6.66E-04	8.27E-03	5.04E-04	1.35E-02
adipocytokine signaling pathway	kegg	8.26E-06	8.49E-05	3.27E-04	5.15E-03	5.16E-04	1.35E-02
IL2 stat5 signaling	hallmarks	5.11E-07	9.27E-06	1.34E-05	3.96E-04	8.71E-04	1.47E-02
glycolysis	hallmarks	2.50E-04	1.52E-03	1.34E-05	3.96E-04	8.71E-04	1.47E-02
TP53 pathway	hallmarks	8.10E-05	5.62E-04	4.45E-04	5.84E-03	8.71E-04	1.47E-02
glycerophospholipid metabolism	kegg	7.61E-04	3.82E-03	6.49E-05	1.39E-03	8.73E-04	1.47E-02
aldosterone signaling	kegg	3.33E-04	1.92E-03	3.45E-05	8.15E-04	1.60E-03	2.51E-02
GNRH signaling pathway	kegg	5.01E-03	1.85E-02	2.10E-03	1.82E-02	2.38E-03	3.51E-02
endocytosis	kegg	2.74E-04	1.62E-03	1.30E-03	1.46E-02	3.50E-03	4.34E-02
glycolysis gluconeogenesis	kegg	1.40E-02	3.62E-02	2.26E-03	1.84E-02	4.85E-03	4.44E-02
interferon gamma response	hallmarks	2.09E-12	4.93E-10	1.34E-05	3.96E-04	5.09E-03	4.44E-02
estrogen response late	hallmarks	8.10E-05	5.62E-04	4.45E-04	5.84E-03	5.09E-03	4.44E-02
inflammatory response	hallmarks	2.00E-03	8.12E-03	4.45E-04	5.84E-03	5.09E-03	4.44E-02
heme metabolism	hallmarks	8.10E-05	5.62E-04	2.15E-03	1.82E-02	5.09E-03	4.44E-02

The analysis is focused on coactivation by KDM3A/AR ChIP and transcriptomic change upon knockdown. Input gene sets were defined by coactivation by KDM/AR ChIP-Seq (1912 genes; marked with *), coactivation by KDM/AR ChIP-Seq and transcriptomic change upon *KDM3A*/*AR* knockdown (421 genes; marked with **), oncogenic gene up-regulation by KDM3A/AR and KDM/AR ChIP coactivation (260 genes; marked with **) (compare Figure 4). Comprehensive lists of gene sets and enrichment ratios are deposited in the Supplementary Information. To determine significance of pathway enrichment thresholds of 0.05 and 0.10 were used for p-values and q-values, respectively according to multiple hypotheses testing using the controlling procedure of Benjamini and Hochberg.

**Table 4:** Epigenetic profiles and transcription factor motifs in H3K9me1/2-KDM ChIP-Seq data of the androgen response pathway

Target gene	KDM TSS	KDM Exon	KDM Intron	KDM TTS	KDM3A regulation	AR regulation
*KRT19*	+	+	+	+	−4.5999	−0.7642
*NKX3-1*	+	-	-	+	−2.8073	−1.0951
*KLK3*	+	+	+	-	−1.3230	−0.5316
*NDRG1*	-	+	+	+	−1.2254	-
*MAF*	+	+	-	-	−1.1679	-
*CREB3L4*	+	+	+	+	−1.1671	-
*MYC*	+	+	+	+	−1.0253	−0.5261
*INPP4B*	+	+	+	+	−1.0252	−0.6637
*PTK2B*	-	+	+	-	−0.7523	−0.4538
*MAPK1*	+	-	+	+	−0.7441	-
*MAP2K1*	+	+	+	+	−0.7212	-
*IGF1*	+	+	+	+	−0.7062	−0.5278
*E2F1*	+	+	-	-	−0.6708	-
*HSP90AA1*	+	-	+	-	−0.6499	-
*HIF1A*	+	+	+	+	−0.6010	-
*ACSL3*	-	+	+	+	−0.5263	−0.8003
*CHUK*	+	-	+	-	0.4407	-
*CDKN1A*	+	-	+	-	0.7347	-

Transcriptomic validation of gene expression change of target gene following knockdown of *KDM3A* or *AR* is listed as LOG2 (fold change).

## DISCUSSION

Epigenetic regulators like KDM3A specifically demethylate histone marks H3K9me1 and H3K9me2, thereby playing a central role in the histone code. In cancer, demethylation and decondensation of chromatin can lead to dysregulated gene expression and transcriptional activation of gene targets [21]. Previous cell biological studies have suggested that KDM3A may stimulate transcription mediated by nuclear receptors and/or that KDM3A may be involved in activation of forkhead proteins during cell differentiation [17, 22]. While histone modifiers are not limited to specific DNA cognate sites, transcriptional specificity can be accomplished by cooperation with transcription factors recognizing distinct DNA motifs [13].

The chosen bioinformatics approach of quantifying changes of matched epigenetic remodeling in combination with transcription factor ChIP-Seq and transcriptomic analysis following knockdown of an epigenetic master regulator offers insight from two angles: it can identify in an unbiased way genome-wide cooperation of epigenetic remodeling with other members of the transcriptional machinery, and it can elucidate details of the interaction. In prostate cancer, steroid ligand dependency or independency can influence the prognostic outcome. The CWR22Rv1 cell line offers the benefit of being able to probe a static, aggressive end point of the disease, while it has limitations due to lacking the dynamic ligand-dependent aspect of AR signaling. Since the CWR22Rv1 cell line expressed permanently activated AR without the ligand binding domain, it provides a stable model for studying the dynamic response to epigenetic regulation by *KDM3A* knockdown in combination with ChIP-Seq analysis. *KDM3A* knockdown abolished tumor formation in an orthotopic prostate tumor model using CWR22Rv1 cells [18]. Interestingly, *KDM3A* knockdown in other prostate cancer cell lines, including the androgen dependent LNCaP cells, blocked cell proliferation [18].

Androgen signaling is subject to multilevel control. In addition to agonist and antagonist ligand chaperones, intracellular localization and interactions with other transcription factors or histone modifiers can influence the transcriptional outcome. Multiple epigenetic regulators have been described to interact with the AR. KDM4C co-localizes with the androgen receptor in prostate carcinomas, and knockdown of *KDM4C* inhibits transcriptional activation and tumor cell proliferation [23]. KDM3A is involved in spermatogenesis by regulating expression of target genes such as *PRM1* and *TMP1*, which are required for packaging and condensation of sperm chromatin [3]. Furthermore, its involvement in obesity resistance through regulation of metabolic genes such as *PPARA* and *UCP1* highlight a transcriptional network focused on lipid modifiers.

In this epigenetic and transcriptomic study we aimed to outline specific pathways of KDM3A demethylase action and enriched transcriptional networks under its control. Previous studies have outlined KDM3A expression levels in prostate cancer phenotypes [24], but KDM3A-regulated target pathways by ChIP-Seq analysis were unknown [24–26]. Motif-guided searches for cooperating transcription factors can link transcriptional programs with genome-wide histone modifications. Motifs of SREBP, HIF, AP1, KLF, MYC, and FOX families are enriched in the H3K9me1/2-KDM ChIP-Seq data and were described to play a role in prostate cancer progression [3, 9, 27–31]. However, of the enriched transcription factors characterized so far, only AR and MYC have strong biochemical links to KDM3A [3, 18]. Solely based on KDM3A regulation of ChIP-Seq and transcriptomic data, a gene set of 1408 genes revealed androgen-related signaling as top hit. Within the androgen response, transcriptionally validated genes, *NKX3-1* and *KLK3*, have been shown to have dynamically regulated histone modification states [32].

In a second step, matched ChIP-Seq studies of KDM3A and the AR allowed us to focus on a distinct transcriptional network. Aside from the androgen response, cellular metabolism is highly enriched in the executioner program of KDM3A and the AR. In particular, lipogenic and hypoxic metabolism stand out for their regulation in the combined KDM3A/AR ChIP-Seq and transcriptomic data. Previously established AR targets involved in cellular metabolism include *ACSL3*, which is involved in converting free long chain fatty acids into fatty acyl-CoA esters [33, 34]. Genome-wide ChIP-Seq profiles show that KDM3A regulates the expression of *FKBP5*, *CHUK*, *HSP90AA1*, and *VHL*. These genes were previously implicated as being regulated by the AR and their proteomic function is involved in AR folding, transactivation, and translocation in the nucleus [35–38]. *HSP90AA1* is described to be under the control of KDM3A [39–41]. Prominent shared targets of KDM3A and AR include gene targets associated with hypoxic metabolism. Transcriptional regulation in response to hypoxia is regulated by the actions of HIF1A and controls glycolytic metabolism [42]. Previously, *KDM3A* gene expression was identified as one of the genes under control of HIF1A [43]. In addition, there is increased transcriptional activity of AR within castration-resistant prostate cancer by hypoxia [44]. A similar overlapping network of KDM3A demethylation, nuclear hormone signaling, and hypoxia is described in estrogen independent breast cancer models [16]. KDM3A's ability to regulate metabolic gene expression by controlling AR binding site availability in hypoxic cells may be the molecular action KDM3A utilizes to stimulate tumor progression [45]. The additional enriched pathways with shared KDM3A and AR regulation appear to the hypoxic cell response. Cytokine and metabolic signaling is able to induce expression of HIF1A and promote its activation in an oncogenic fashion [46–51].

The functional impact of coordinated action between a lysine demethylase and transcription factors may lend to its target specificity, or at the very least, create accessibility for DNA binding [52–56]. KDM3A controlled H3K9me1/2 ChIP-Seq data shows a strong enrichment of AR binding sites within the CWR22Rv1 castration-resistant prostate cancer cell line with continued expression of genes involved in the androgen response. However, about a third of the AR ChIP-Seq peaks were suppressed in response to increased H3K9me1/2 signal in the KDM3A experiments. It remains to be determined which other coactivators and corepressors take charge of AR binding sites in genomic locations where KDM3A has no effect on AR binding or transcriptional response. Notably, selected KDM3A- or AR-dependent genes show no clear association of modulated H3K9me1/2 marks within 50,000 bp around the gene body, suggesting that KDM3A can regulate gene expression either by accessing distant enhancers or by physical interaction with the transcriptional machinery independent of H3K9 demethylation. For example, KDM3A was identified to erase lysine 372 monomethylation of TP53, a protein methylation site crucial for the stability and pro-apoptotic function of chromatin-bound tumor suppressor [57]. Significantly, next to the AR, SREBF, and HIF, the conducted transcription factor motif analysis confirmed a strong presence of TP53 target sequences controlled by KDM3A. Extensive future ChIP-Seq studies of KDM3A as well as other candidate transcription factors associated with KDM3A will be necessary to further characterize the full spectrum of epigenetic and transcriptional control of the master regulator KDM3A.

In conclusion, the ChIP-Seq study refined the genomic sites of KDM3A-mediated H3K9me1/2 histone demethylation within the CWR22Rv1 prostate cancer cell line. The cancer systems biology analysis expanded underlying transcription factor motifs associated with oncogenic KDM3A demethylation, suggesting an underlying transcriptional network that directs transcriptional activation. Future experimental verification of epigenetic hotspots is needed to determine when detected response elements are functional in gene regulation. The matched transcriptomics and epigenomics approach identified an overlap between androgen receptor ChIP-Seq and KDM3A-regulated H3K9me1/2 ChIP-Seq. The comprehensive genome-wide mapping of matched ChIP-Seq profiles highlighted mechanistic details of how an epigenetic master regulator can exhibit control over selected transcriptional programs, such as metabolic pathways and hypoxia response in cancer.

## MATERIALS AND METHODS

### Tissue culture of prostate cancer cell lines

CWR22Rv1 is a human prostate carcinoma epithelial cell line derived from a xenograft that was serially propagated in mice after castration-induced regression and relapse of the parental, androgen-dependent CWR22 xenograft [58, 59] (CRL-2505, American Type Culture Collection, Manassas, VA). The CWR22Rv1 prostate cancer cell line was kindly provided by Dr. James Jacobberger (Case Western Reserve University, Cleveland, OH), and are maintained in RPMI 1640 medium supplemented with 10% fetal bovine serum and antibiotics. Cell cultures are regularly tested to ensure absence of mycoplasma. The CWR22Rv1 prostate cancer cell line expresses AR full-length with a duplicated DNA binding domain in exon 3 and AR splice variants, for example AR-v7, lacking a ligand binding domain. Thus, the CWR22Rv1 cell line displays constitutively active AR even in the absence of androgen [59]. In the ChIP-Seq study an AR antibody (PG21, 06-680, Sigma EMD Millipore, Darmstadt, Germany) was used recognizing both forms of the AR. All experimental protocols were approved by the Institutional Review Board at the University of California Merced. The study was carried out as part of IRB UCM13-0025 of the University of California Merced and as part of dbGap ID 5094 on somatic mutations in cancer and conducted in accordance with the Helsinki Declaration of 1975.

### Knockdown of KDM3A with shRNA

Lentiviral vectors encoding *KDM3A* small hairpin RNA (shRNA), *AR* shRNA or lentiviral pLKO.1 control shRNA were purchased from Open Biosystems (GE Healthcare Dharmacon, Lafayette, CO), and packaged in human embryonic kidney 293T cells (CRL-3216, American Type Culture Collection, Manassas, VA) by calcium phosphate transfection. The supernatant containing lentiviral particles were collected 48 hours after transfection. CWR22Rv1 cells were transduced with the supernatant of lentiviral particles in the presence of polybrene (8 μg/ml) for 24 hours before replacement with the fresh growth media. Cells were analyzed at 48 hours post-transduction. The knockdown efficiency was confirmed by quantitative real time polymerase chain reaction (qRT-PCR) and Western-blot analysis.

### qRT-PCR analysis

Total RNA from prostate cancer cells was extracted using a mammalian RNA mini preparation kit (RTN10-1KT, GenElute, Sigma EMD Millipore, Darmstadt, Germany) and then digested with deoxyribonuclease I (AMPD1-1KT, Sigma EMD Millipore, Darmstadt, Germany). Complementary DNA (cDNA) was synthesized using random hexamers. Triple replicate samples were subjected to SYBR green (SYBR green master mix, Qiagen SABiosciences) qRT-PCR analysis in an Eco system (Illumina, San Diego). Gene expression profiles were analyzed using the ΔΔCT method. qRT-PCR threshold cycle (CT) values were normalized using the housekeeping gene cyclophilin A (*PPIA*; peptidylprolyl isomerase A; Gene ID: 5478). The following primers served for ChIP-qRT-PCR validation of ChIP-Seq signal of H3K9me1/2-KDM around the AREs of AR target genes: *KLK3* (kallikrein related peptidase 3; Gene ID: 354; also known as PSA, prostate specific antigen): 5′-TGGGACAACTTGCAAACCTG-3′; 5′-CCAGAGTAGGTCTGTTTTCAATCCA-3′; *NKX3.1* (NK3 homeobox 1; Gene ID: 4824): 5′-GGTTCTGCTGTTACGTTTG-3′; 5′-CTTGCTTGCTCAGTGGAC-3′; *MYC* (v-myc avian myelocytomatosis viral oncogene homolog; Gene ID: 4609): 5′-CCAGCGAATTATTCAGAA-3′; 5′-AATTACCATTGACTTCCTC-3′.

### Western-blot analysis

Whole cell lysates were harvested using radio-immunoprecipitation assay (RIPA) buffer composed of 50 mM trisaminomethane hydrochloride (Tris-HCl) pH7.5, 150 mM sodium chloride (NaCl), 1% Triton X-100, 0.1% sodium dodecyl sulfate (SDS), 0.1% sodium deoxycholate, 1.0 mM EDTA, 1.0 mM sodium orthovanadate, and 1x protease inhibitor cocktail. Lysates were subjected to sodium dodecyl sulfate-polyacrylamide gel electrophoresis (SDS-PAGE) and proteins transferred to a nitrocellulose membrane (GE Healthcare Life Sciences, Pittsburgh, PA). The membrane was probed with ChIP-grade H3K9me1 (ab9045, Abcam, Cambridge, MA), H3K9me2 (07-441, Sigma EMD Millipore, Darmstadt, Germany), KDM3A (A301-539A, Bethyl Laboratories, Montgomery, TX), or ACTB (A5441, Sigma EMD Millipore, Darmstadt, Germany) antibodies followed by a secondary antibody conjugated to fluorescent dye, and blots were imaged using the odyssey detecting system (LI-COR Biotechnology, Bad Homburg, Germany).

### Chromatin immunoprecipitation

Cells were crosslinked using 1% formaldehyde for 10 min at 298 K. Formaldehyde was removed and cells were incubated with 125 mM glycine for 5 min at 298 K. Nuclear extracts were collected and sonicated to obtain 300 bp chromatin fragments using the Covaris S2 ultrasonicator (Covaris, Woburn, MA). 100 μg of chromatin was incubated with 5 μg of AR (PG21, 06-680, Sigma EMD Millipore, Darmstadt, Germany), 5 μg of KDM3A (A301-538A, Bethyl Laboratories, Montgomery, TX), 2 μg of H3K9me1 (ab9045, Abcam, Cambridge, MA), or 2 μg of H3K9me2 (07-441, Sigma EMD Millipore, Darmstadt, Germany) antibodies overnight at 277 K followed by incubation with 30 μl of protein A/G beads for 4 hours. After four washes, crosslinking was reversed. Chromatin was digested with ribonuclease A followed by proteinase K. Then the DNA was purified using spin columns. The size of the DNA was confirmed by a bioanalyzer (Agilent Biotechnologies, Savage, MD).

### Next generation sequencing and ChIP-Seq analysis

The purified DNA library was sequenced using an Illumina HighSeq2000 at the Sanford-Burnham Medical Research Institute at Lake Nona, National Genome Library Core Facility. Sequenced regions were aligned to the reference human genome 19 using the Bowtie alignment program that utilizes an extended Burrows-Wheeler indexing for an ultrafast memory efficient alignment [60]. Peak calling utilized a model-based analysis of ChIP-Seq (MACS) algorithm [61, 62]. The overlap analysis, plot of genomic location, sequence extraction, motif identification, and peak filtering were performed using ChIPseek: a web-based analysis for ChIP data [63]. ChIPseek also employs scripts from BEDtools [64] using a genome binning algorithm used by the UCSC genome browser to sort genomic regions into groups along the length of chromosome [65]. Data visualization was carried out using the integrative genomics viewer [66].

### Motif analysis based on position site-specific matrix models

Computational response element searching algorithms are able to estimate a sequence's likelihood in belonging to the response element of the query transcription factor using position site-specific matrix (PSSM) models where each position in the query transcription factor model gives each of the four letters in the DNA alphabet a score based on the probability of that nucleotide being found at that position [67]. Summation into a logs-odd score is converted into a p-value assuming a zero-order background model, and all response elements less than the threshold are reported [68]. Motif discovery, motif enrichment, and motif scanning used the multiple expectation maximization for motif elicitation (MEME) and discriminative regular expression motif elicitation (DREME) suite software toolkits from a set of user supplied unaligned sequences for ChIP-Seq regions [69]. *De novo* motif analysis programs MEME and DREME identify frequently detected DNA sequences patterns and similarity matches of recurring ChIP-Seq sequences with DNA motifs of deposited studies in genomic sequence databases [68, 70]. After a motif of interest is discovered the genomic sequences of the ChIP sequenced data is scanned using the MEME suite software find individual motif occurrences (FIMO) [68] for individual motif occurrences using PSSMs to compute a log-likelihood ratio score for each submitted sequence. Sequence-specific matrix models are further used to analyze the next generation sequencing data for motif enrichment and potential coactivators [13, 71].

### Microarray analysis

In order to quantify the transcriptomic effect of KDM3A or AR knockdown, a microarray profiling analysis was conducted on CWR22Rv1 knockdown cells. CWR22v1 cells were transduced with lentiviral pLKO.1 control shRNA vector, *AR*, or *KDM3A* shRNA for 48 h. Total RNA was isolated from cells, and 500 ng was used for synthesis of biotin-labeled cRNA using an RNA amplification kit (Ambion, Thermo Fisher Scientific, Waltham, MA). Biotinylated cRNA was labeled by incubation with streptavidin-Cy3 to generate a probe for hybridization with the GeneChip Human Transcriptome Array 2.0 (Affymetrix Inc, Santa Clara, CA) or the genome-wide transcriptome Human HT-12 V4.0 (Illumina Inc, San Diego, CA). Four samples from two experimental groups (n=2 per group) were hybridized to the chip to obtain raw gene expression data, which was processed to obtain raw data in the form of expression intensities. Raw data was then exported for further processing and analysis using R statistical software version 3.1 in combination with the BioConductor oligo, affy and genefilter packages [72]. The raw signal intensities were background corrected, LOG2 transformed, and quantile normalized to generate robust multi-array average (RMA) normalized intensities [73]. Quality control analyses did not reveal any outlier samples. Differential expression between experimental groups was assessed by generating relevant contrasts corresponding to the two-group comparison and was evaluated using the Linear Models for Microarray Analysis (LIMMA) package [72, 74]. Raw p-values were corrected for multiple hypotheses testing using the false discovery rate controlling procedure of Benjamini and Hochberg, and adjusted p-values below 0.05 were considered significant [75]. Genes with significantly altered expression levels with adjusted p-values below 0.05 following *KDM3A* knockdown were selected and analyzed through the use of Ingenuity Pathway Analysis (IPA, Qiagen, Redwood City, CA). Pathway enrichment of differentially expressed genes was determined by gene set enrichment analysis (GSEA) by pairing each gene with its expression value and ranking genes in descending order (Broad Institute, Cambridge, MA) [76, 77]. As we are testing multiple gene sets at the same time, the p-values need to be controlled for false positives. The false discovery rate estimation for the pathway enrichment is as summarized in q-values with a threshold of 0.10 controlling the error rate and correcting for multiple hypotheses testing according to Benjamini and Hochberg [75]. Acquired data of transcriptome profiling microarray analysis of CWR22Rv1 cells with *AR* knockdown using GeneChip Human Transcriptome Array 2.0, platform GPL16686 (Affymetrix Inc, Santa Clara, CA), is deposited in the NCBI GEO database under accession number GSE86547. Acquired data of CWR22Rv1 cells with *KDM3A* knockdown using hybridization with genome-wide transcriptome Human HT-12 V4.0, platform GPL10558 (Illumina Inc, San Diego, CA), is deposited under accession number GSE70498.

### Availability of supporting data

The Supplementary Information contains tables on genome-wide mapping, annotation, and overlap of H3K9me1/me2 demethylation ChIP-Seq and AR ChIP-Seq (Supplementary Table 1), gene sets based on ChIP-Seq and transcriptomic data (Supplementary Table 2), pathway enrichment analysis based on H3K9me1/me2 demethylation ChIP-Seq gene set (Supplementary Table 3), transcriptomic response upon shRNA(*KDM3A*) and shRNA(*AR*) knockdown (Supplementary Table 4), hierarchical gene set enrichment analysis of identified KDM3A target genes (Supplementary Table 5), and an upstream regulator analysis based on ingenuity pathway analysis (Supplementary Table 6).

## SUPPLEMENTARY TABLES




