# Supplementary material for: The histone demethylase KDM3A regulates the transcriptional program of the androgen receptor in prostate cancer cells
Source: Oncotarget. 2017 Mar 3;8(18):30328–43. doi: 10.18632/oncotarget.15681 (PMC5444746; doi:10.18632/oncotarget.15681)
Supplement: Supplementary file 1 [file oncotarget-08-30328-s001.pdf]

The histone demethylase *KDM3A* regulates the transcriptional program of the androgen receptor in prostate cancer cells

SUPPLEMENTARY TABLES

Supplementary Table 1: Genome-wide mapping, annotation, and overlap of H3K9me1/me2 demethylation ChIP-Seq and AR ChIP-Seq

See Supplementary File 1

Supplementary Table 2: Gene sets based on ChIP-Seq and transcriptomic data

See Supplementary File 1

Supplementary Table 3: Pathway enrichment based on H3K9me1/me2 demethylation ChIP-Seq gene set

See Supplementary File 1

Supplementary Table 4: Transcriptomic response upon shRNA(*KDM3A*) and shRNA(*AR*) knockdown

See Supplementary File 1

Supplementary Table 5: Hierarchical gene set enrichment analysis of identified KDM3A target genes

See Supplementary File 1

Supplementary Table 6: Upstream regulator analysis based on ingenuity pathway analysis

See Supplementary File 1
